# Integrating multi-omics to unravel host-microbiome interactions in inflammatory bowel disease

**DOI:** 10.1016/j.xcrm.2024.101738

**Published:** 2024-09-17

**Authors:** Yiran Zhang, John P. Thomas, Tamas Korcsmaros, Lejla Gul

**Affiliations:** 1 Department of Surgery & Cancer, Imperial College London, London W12 0NN, UK; 2 Department of Metabolism, Digestion and Reproduction, Imperial College London, London W12 0NN, UK; 3 UKRI MRC Laboratory of Medical Sciences, Hammersmith Hospital Campus, London W12 0HS, UK; 4 NIHR Imperial BRC Organoid Facility, Imperial College London, London W12 0NN, UK; 5 Quadram Institute Bioscience, Norwich Research Park, Norwich NR4 7UQ, UK

## Abstract

The gut microbiome is crucial for nutrient metabolism, immune regulation, and intestinal homeostasis with changes in its composition linked to complex diseases like inflammatory bowel disease (IBD). Although the precise host-microbial mechanisms in disease pathogenesis remain unclear, high-throughput sequencing have opened new ways to unravel the role of interspecies interactions in IBD. Systems biology—a holistic computational framework for modeling complex biological systems—is critical for leveraging multi-omics datasets to identify disease mechanisms. This review highlights the significance of multi-omics data in IBD research and provides an overview of state-of-the-art systems biology resources and computational tools for data integration. We explore gaps, challenges, and future directions in the research field aiming to uncover novel biomarkers and therapeutic targets, ultimately advancing personalized treatment strategies. While focusing on IBD, the proposed approaches are applicable for other complex diseases, like cancer, and neurodegenerative diseases, where the microbiome has also been implicated.

## Graphical abstract

**Figure:**
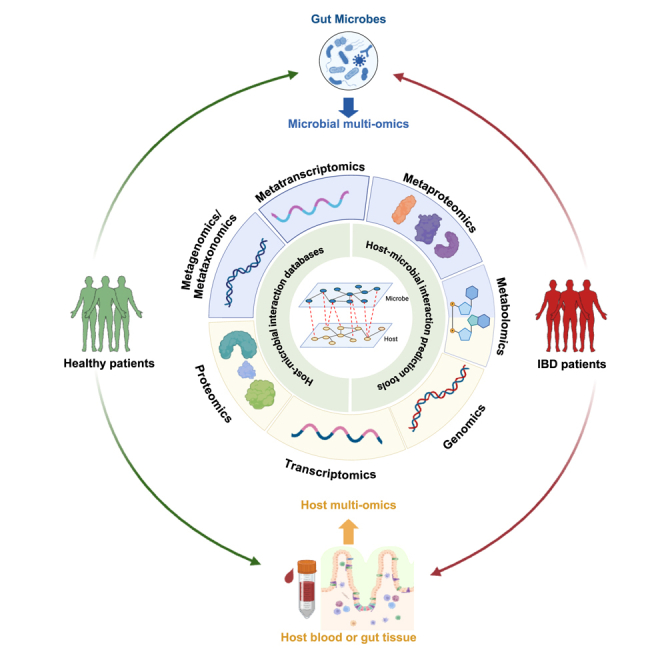

## Teaser

This review discusses the impact of multi-omics technologies in exploring the complex interplay between gut microbiota and host cell responses in inflammatory bowel disease (IBD). By integrating microbiome and host-omics data, this review provides insights into the molecular mechanisms underlying IBD pathogenesis and facilitate the development of personalized therapeutic strategies.

## Introduction

The human microbiome comprises a diverse community of microorganisms including bacteria, virus, fungi, and archaea, which exists in symbiosis with the human host and plays a crucial role in maintaining homeostasis. This is particularly evident in the gastrointestinal (GI) tract, where the gut microbiome plays an essential role in facilitating nutrient and drug metabolism, maintaining the gut epithelial barrier, and regulating the immune system.^1^ Disruption in the gut microbiome, known as dysbiosis, has been closely associated with diseases, like inflammatory bowel disease (IBD).^2^

IBD represents a heterogeneous group of chronic inflammatory conditions affecting the GI tract, with Crohn’s disease (CD) and ulcerative colitis (UC) being the two major subtypes.^3^ IBD usually onsets in early adulthood, causing substantial morbidity with symptoms such as bloody diarrhea, abdominal pain, and weight loss.^4^ Despite advanced therapies (biologic and small-molecule agents) targeting dysregulated immune pathways, only a minority of patients (∼30%) achieve sustained clinical remission,^5^ highlighting the need for a better understanding of IBD pathogenesis.

IBD is thought to arise due to complex interactions between multiple genetic risk factors and environmental insults, ultimately resulting in gut dysbiosis and immune system overactivation.^3^ Gut dysbiosis is critical to IBD pathogenesis, as evidenced by the increasing incidence of the disease in newly industrialized countries.^6^^,^^7^ The “hygiene hypothesis” has been proposed to explain this phenomenon that reduced childhood exposures to GI pathogens, and increased antibiotics treatments have led to a less tolerogenic gut immune system, which is more susceptible to inflammation.^8^ Mechanistic insights from mouse models of colitis complement these epidemiological observations. Germ-free conditions ameliorate inflammation in various colitis models (e.g., *IL10*^*−/−*^ mice), and fecal microbiota transplantation from dysbiotic *T-bet*^*−/−*^ *RAG2*^*−/−*^ UC mice induces colitis in wild-type mice.^9^ These findings demonstrate the importance of the gut microbiota for the development of colitis in both innate and adaptive murine models of IBD. In humans, surgical diversion of the fecal stream reduces the risk of disease recurrence in patients with CD,^10^ while exclusive enteral nutrition, which results in significant changes to the gut microbiota, is an effective strategy for inducing remission.^11^ Alternatively, more tolerable strategies (including fecal microbiota transplantation and probiotic therapies) have demonstrated variable efficacy, and as such are not currently used in clinical practice.^12^^,^^13^ This reflects a major gap in our current understanding of the specific host signaling pathways modulated by the gut microbiome, both in health and in the context of IBD.

Traditional methods for studying host-microbial interactions (HMIs)—such as culture-dependent analysis of the microbiota or analyzing a single biological level (e.g., gene expression or protein abundance)—have provided valuable insights, but these methods did not capture the full complexity of these interactions.^14^ The advent of next-generation sequencing and high-throughput technologies, like metagenomics, metabolomics, and proteomics, has transformed our ability to systematically map the molecular constituents of these intricate systems.^15^ Harnessing multiple omics technologies simultaneously offers an unprecedented opportunity to reveal molecular compositions and dissect HMIs in IBD pathogenesis. Metagenomic and metataxonomic sequencing identify microbial species and their genetic presence in the samples. Metaproteomics reveals the protein content and indicates the functionality of the community.^14^ Metabolomics unveils the small molecule composition identifying metabolites that can originate from the host, microbes, or diet. Among these, small molecules with immunomodulatory potential, such as short-chain fatty acids and bile acids, play significant roles in IBD. Throughout this review, we acknowledge both host-derived and microbial-derived metabolites. Nevertheless, some resources or tools may not clearly specify the origin, referring to either or both sources of metabolites.

While single- and more recently multi-omic datasets from patients with IBD are increasing, discovering the complex interplay between host and microbes in disease remains a challenge.^16^ Systems biology, the systematic study of complex interactions within biological systems, has emerged as a powerful and versatile approach to reveal holistic mechanisms underpinning HMIs in IBD, which may not be captured through single-omics analysis alone.^17^ The reusability of multi-omics data, combined with advanced modeling techniques, facilitates comprehensive and integrative analyses of these complex systems. Publicly available datasets facilitate the reuse of high-quality data, while machine learning and network analysis tools can uncover patterns and relationships within them. Integrative multi-omics approaches, such as multi-omics factor analysis and systems biology modeling, allow for the simultaneous analysis of multiple biological layers, revealing cross-omics correlations and causal relationships. In the context of IBD, these methods have been used to identify biomarkers, understand disease heterogeneity, and pinpoint therapeutic targets, with longitudinal modeling providing dynamic insights into disease progression and treatment responses. Despite challenges like high inter-individual variability and the need for improved spatial resolution, advancements in statistical models and technology sensitivity are paving the way for more detailed and accurate simulations of cellular and molecular dynamics, ultimately enhancing our understanding and treatment of complex diseases.^18^

Spurred by the omics revolution, numerous systems biology tools and resources have been developed in recent years, but these have yet to penetrate the field of IBD. These novel resources represent a major opportunity to harness existing and newly generated omics datasets to gain novel insights into IBD pathogenesis. This review aims to provide an overview of the latest systems biology tools and resources that could be utilized to gain deeper insights into HMIs through multi-omics data integration.

### Multi-omics host-microbiome projects in IBD

With the increasing accessibility to omics technologies, there are now a growing number of studies employing multi-omics profiling to simultaneously chart perturbations in both host tissues and the gut microbiome in patients with IBD.

Among these, the study by Lloyd-Price et al.,*^19^* part of the Integrative Human Microbiome Project in the US, collected longitudinal multi-omics data from 110 patients with IBD (CD = 68, UC = 38) and 27 non-IBD controls over the course of 1 year, including from intestinal tissues (bulk transcriptomics, epigenetic reduced representation bisulfite sequencing [RRBS], and 16S rRNA gene amplicon sequencing), stool samples (metagenomics, metatranscriptomics, proteomics, and metabolomics), and peripheral blood (human exome sequencing, serology, and RRBS). These data, available in the open-access IBD Multi’omics Database (IBDMDB) (https://ibdmdb.org/),^19^ identified dysbiotic samples in patients with IBD, characterized by an enrichment of facultative anaerobes, such as *Escherichia coli*, and a depletion of beneficial species (*Roseburia hominis*, and *Ruminococcus torques* and *Ruminococcus gnavus*). Integrative analysis revealed correlations between the various -omic layers highlighting that *Faecalibacterium prausnitzii* had strong correlations with numerous downregulated microbial enzymes in dysbiosis, while *Escherichia coli* had strong associations with upregulated microbial enzymes. *Roseburia* genus members were linked to bile acids and acylcarnitines, suggesting their role in the carnitine and bile acid dysregulation in IBD. Additionally, negative associations were identified between chemokines (e.g., CXCL6 and CCL20) and certain bacteria like *Eubacterium rectale*, *Streptococcus*, and *Eikenella*. Although pioneering in using longitudinal multi-omics profiling to identify differences between patients with IBD and non-IBD controls, the study was limited by a heterogeneous population and simple correlation methodology, which failed to reveal mechanistic insights regarding specific HMIs contributing to IBD pathogenesis.

Other studies have recently identified treatment response signatures using multi-omics profiling. One of the largest studies to date was by Lee et al.,^20^ which profiled baseline stool (metagenomics) and serum (metabolomics and proteomics) of patients with active CD (*N* = 108) or UC (*N* = 77) prior to initiating anti-integrin or anti-cytokine (anti-TNF or anti-IL12/23) therapies. This study demonstrated that multi-omics predictors were superior to clinical or single-omic predictors in identifying patients with IBD who respond to advanced therapies. Despite this, the study faced limitations, including the limited statistical power of the analysis and lack of replication cohort, as well as tissue transcriptomics data resulting in inadequate HMI analysis. Larger studies, like IBD-RESPONSE, aim to further define predictive multi-omics biomarkers for treatment response.^21^

Multi-omics analysis is also used to predict the future onset of IBD. One of the largest studies to explore this to date was the Crohn’s and Colitis Canada Genetic Environmental Microbial (GEM) project (https://www.gemproject.ca/).^22^ The GEM project investigated the interplay between genetic, environmental, and microbial factors in CD by prospectively studying healthy first-degree relatives of patients with CD. Baseline fecal 16S RNA metataxonomic and metabolomic profiling were performed on participants, followed prospectively for a median time of 5.4 years during which 73 individuals developed CD.^22^ Using a random forest classifier, investigators developed and validated a microbial risk score (MRS) predicting CD risk, identifying key taxa, such as *Ruminococcus torques*, *Blautia*, *Colidextribacter*, an uncultured genus-level group from *Oscillospiraceae*, and *Roseburia*. Integration of the metagenomic and metabolic profiles revealed negative correlations with anti-inflammatory metabolites such as biotin (vitamin B7) and niacin (vitamin B3) and a positive correlation with pro-inflammatory sphingolipids. The study also evaluated gut barrier function through the urinary fractional excretion of lactulose-to-mannitol ratio to estimate gut barrier function and identified a potential link between gut microbiome compositions, linking microbiome composition to gut barrier impairment.^23^ Dietary analysis showed that a Mediterranean-like diet positively influences microbial composition and reduces gut inflammation.^24^ Furthermore, proteomic profiling identified 25 serum proteins, including CXCL9, associated with future CD development.^25^ Despite novel insights, the predictive capabilities of the MRS were modest and multi-omics integrative analyses as well as mechanistic insights were limited. Large prospective multi-omics inception cohort studies are currently underway to address some of these limitations, such as the recently launched OPEN-IBD (https://www.sanger.ac.uk/collaboration/open-ibd/), which focuses on established IBD and may address limitations of the Integrative Human Microbiome Project (see the text described earlier), while the GEM project aims to identify biomarkers predicting IBD development in healthy first-degree relatives.

In summary, multi-omics approaches are increasingly being utilized to better understand host-microbial relationships in IBD pathogenesis, therapeutic response, and disease onset. Early studies have highlighted challenges such as integrating multi-omics data to pinpoint specific HMIs that could be targeted for therapeutic intervention. Advanced systems biology databases and computational tools are rapidly evolving to address these challenges and have been successfully harnessed in other fields, notably cancer.^26^ These tools can integrate various omics layers, providing insights that single-omics approaches cannot. The following sections will explore these resources and their potential to unlock the full benefits of multi-omics data for understanding IBD pathogenesis and developing more targeted, personalized therapeutic strategies. With major multi-million dollar multi-omics projects currently underway in IBD (e.g., IBD-RESPONSE^21^ and OPEN-IBD), it is critical that such efforts yield translatable insights.

### Systems biology resources to study the impact of the microbiome on the host in IBD

#### Databases for host-microbe interaction research

**Figure 1:**
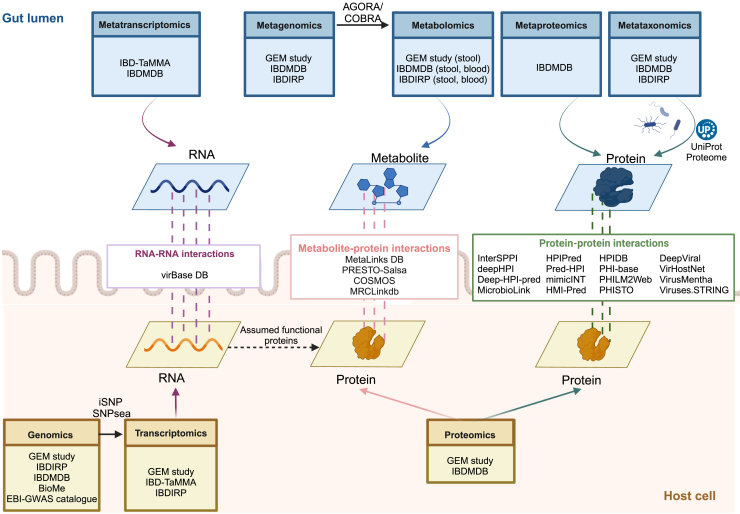
Integration of multi-omics data using various systems biology tools and databases The figure shows example resources highlighted in this review and does not extensively list all IBD resources. The figure was created by BioRender.

The exponential growth of meta-omics and host-omics data necessitates integrating these high-throughput data to investigate the dynamic interplay between the host and microbes better. HMI databases collect experimentally validated protein-protein and metabolite-protein interactions between the human host and various microbes (Figure 1). Such databases can support multi-omics data integration efforts in IBD and other complex diseases by providing evidence for inferring causal associations between microbial and host molecules.

### Protein-protein interaction resources

Microbial proteins significantly impact host signaling pathways, contributing to cellular homeostasis, immune system modulation, and gut barrier functions.^27^ In homeostatic conditions, the gut epithelial layer is covered by a protective mucin layer, which limits direct host-microbial protein interactions.^28^ However, inflammation or mucosal barrier disruption in IBD allows microbial proteins to cross this barrier and interact directly with host proteins.^29^ Currently, understanding the impact of these protein-protein interactions (PPIs) in IBD is limited and needs further investigation.

PPI resources can help researchers to combine metaproteomics (microbial protein data) and host proteomics (host protein data) to predict how microbial proteins impact host cell signaling, offering an integrated view of molecular mechanisms involved in IBD.^30^ Due to technical complexity, extensive sample need, and high cost, these omics datasets are often unavailable. An alternative is integrating metataxonomics and host transcriptomics to explore host-microbe PPIs. Metataxonomics identifies prevalent microbial species in IBD, combining this with the UniProt Proteome database,^31^ the corresponding microbial proteomes could be acquired. Meanwhile, host transcriptomics infers which proteins could be expressed from gene expression data, thereby predicting potential interactions. Several comprehensive resources for host-pathogen interactions (HPIs) are available (Table 1). HPIDB^32^ (https://hpidb.igbb.msstate.edu/) is a curated resource with 69,787 unique protein interactions between viral, bacterial, and fungal pathogens and 70 different hosts, including human, animal, and plant, derived from scientific literature and 12 external resources. Users can search the database by sequence, keyword, and homologous HPIs, and data can be downloaded in standardized PSI-MITAB file format. PHI-base^33^ (http://www.phi-base.org/) catalogs experimentally verified pathogenicity, virulence, and effector genes from fungal, oomycete, and bacterial pathogens, which infect human, animal, plant, fungal, and insect hosts. Compared to HPIDB, this contains fewer interactions due to excluding virus information and focusing on experimentally verified HPIs. PHILM2Web^34^ contains HPIs derived from PubMed abstracts using a literature-mining algorithm. Although PHISTO^35^ (https://phisto.org/) has been published in 2013, it is still an essential resource for studying the impact of viral, bacterial, and fungal pathogens in human,^36^ as it provides experimentally verified interactions integrated from nine databases, with multiple analysis tools.

**Table 1:** Molecular interaction databases to discover host-microbe interactions

*Database*	*Description*	*Number of entries*	*Data sources*	*Last update*
**Host-pathogen interactions**				

*HPIDB 3.0*	Curated host-pathogen interaction data	69,787 PPIs (66 host and 668 pathogen species)	Experimentally verified interactions from IntAct, MINT, UniProtKB, Molecular Connections, MBInfo, I2D, MPIDB, InnateDB, BioGRID, BIND, DIP, MatrixDB, and VirHostNet	July (2016)
*PHI-base 5.0*	Experimentally verified pathogenicity, virulence, and effector genes	27,974 PPIs (220 host and 275 pathogen species)	Experimental evidence from a peer-reviewed publications	March (2024)
*PHILM2Web*	Literature-mining platform for host-pathogen interaction data from PubMed abstracts	23,581 PPIs (157 host and 403 pathogen species)	Literature mining	June (2022)
*PHISTO*	Comprehensive bioinformatics platform for experimentally verified pathogen-human interactions	48,615 PPIs (588 pathogen species)	Experimentally verified interactions from APID, IntAct, DIP, MINT, iRefIndex, STRING, MPIDB, BIND, and Reactome	May (2013)

**Virus-host interactions**				

*ViRBase v3.0*	Virus-host ncRNA-associated interactions	827,105 RNA-RNA interactions (116 virus and 36 host species)	Experimental (PubMed, VIRmiRNA, and VmiReg) and predicted (Human-ViCe, RepTar, VmiReg, and Zikv-CDB) interactions	August (2021)
*VirHostNet 3.0*	Network-based exploration of virus-host PPIs	55,115 virus-host and virus-virus PPIs	Literature curation and high-confidence third party databases (IntAct, MINT, DIP, InnateDB, BIND, UniProt, and HPIDB)	March (2022)
*VirusMentha*	Virus-host PPI collection	15,967 PPIs between 5,828 proteins from 12,765 publications	Literature mining and curation by IMEx databases (MINT, IntAct, DIP, MatrixDB, and BioGRID)	June (2024)
*Viruses.STRING*	Virus-host and virus-virus PPI collection	177,425 PPIs between 239 viruses and 319 hosts	Experimental data from BioGrid, MintAct, DIP, HPIDB, and VirusMentha; orthologous relationship transfer; text-mining evidence using the STRING text mining pipeline	July (2023)

**Metabolite-related databases**				

*MetalinksDB*	Comprehensive database for intercellular metabolite-protein interactions.	10,165 metabolite-receptor interactions	STITCH, HMDB, Recon3D, TransportDB, GEMs, NeuronChat, and CellphoneDB	January (2024)
*MiMeDB 1.0*	Detailed information about metabolites discovered in the human microbiome.	24,254 metabolites from 1,904 microbial taxa	VMH, HMDB, KEGG, GenBank, UniProt, BacMap, and FooDB	August (2022)
*PRESTO-Salsa*	Multiplexed screening of metabolome-GPCRome interactions atlas	Interactions between 1,041 metabolites and 314 GPCRs	Comprehensive screening of over 1,000 metabolites and 435 microbiome strains against nearly all human GPCRs	June (2023)

Studying bacterial disequilibrium in patients with IBD can be enhanced by combining datasets with competition assays or co-culture experiments focusing on microbiome, epithelial cells, and immune cell interactions. Notable resources include the NCBI Gene Expression Omnibus^37^ that hosts datasets from numerous studies, including co-culture experiments relevant to IBD; and the IBDMDB**,** which provides multi-omics data to study host-microbiome interactions (details aforementioned). Integrating these databases adds another layer to multi-omics systems biology approaches, offering deeper insights into the complex interplay within the gut environment in IBD.

Although many HPI databases cover bacterial and fungal species, there is a lack of resources describing commensal bacteria-human PPIs. Understanding these interspecies interactions can provide important health insights, but benefits are often indirect and long term leading to prioritization of pathogenic species in PPI data generation. The human microbiome consists of diverse bacterial species, each with unique proteins, making mapping PPIs in such a dynamic environment technically challenging and resource-intensive. The “tools to explore host-microbe interactions” section introduces software for identifying host-bacteria interactions beyond pathogens.

There is growing evidence that gut viral communities (i.e., the gut virome), which coexist with other microorganisms within the gut microbiome, are dysregulated in patients with IBD.^38^ Literature demonstrates that gut viruses are associated with the development of IBD by influencing intestinal immunity and barrier function.^38^ Multiple databases have been established to connect metaproteomics/viromics and proteomics resources, providing a better understanding of virus infections. Resources like VirHostNet^39^ (https://virhostnet.prabi.fr/), VirusMentha^40^ (https://virusmentha.uniroma2.it/), and Viruses.STRING^41^ (http://viruses.string-db.org/) have been published to gather knowledge about virus-host PPIs. VirHostNet is one of the earliest databases containing host-virus PPIs and is still regularly updated (last update in 2022). VirusMentha is an extension of VirusMINT^42^ (published by the same research group), containing virus-virus and host-virus PPI data, updated regularly by capturing curated interactions from external databases. Viruses.STRING is an extension of the popular PPI database, STRING,^43^ combining both experimental and orthologous relationship transfer evidence for inferring host-virus PPIs describing confidence scores that represent the likelihood of interactions. Researchers can use these databases to identify critical host-virus interactions specific to IBD. Cross-referencing the known host-virus interactions with IBD-specific omic datasets enables a comprehensive investigation on how specific viruses might interact with dysregulated host proteins in IBD.

### RNA-RNA interaction resources

Increasing evidence suggests that host RNAs, especially non-coding RNAs (ncRNAs), such as microRNAs (miRNAs) and circular RNAs (circRNAs), modulate disease phenotypes in response to the gut microbiome.^44^^,^^45^ miRNAs (20–25 nucleotides) play essential roles in post-transcriptional gene regulation and are important in IBD pathogenesis influencing inflammation and intestinal barrier function.^46^ circRNAs, single-stranded and covalently closed RNA molecules, influence IBD development and are potential biomarkers and therapeutic targets.^47^ Furthermore, RNA molecules from the gut virome can potentially interact with host RNAs, impacting host signaling. Integrating metatranscriptomics and transcriptomics data with RNA-RNA interaction resources provides insights into how these molecules regulate inflammation and immune responses in IBD. These multi-layered networks have the potential to contribute to the development of RNA-based interventions in IBD.^48^ ViRBase^49^ (http://www.virbase.org/) focuses on ncRNA interactions between viruses and hosts, containing 827,105 virus-host ncRNA-associated interaction entries with annotations (e.g., RNA annotations, single-nucleotide polymorphism [SNP], and drug-associated information) and binding site prediction tools (IntaRNA^50^ and PRIdictor^51^). ViRBase integrates experimentally validated and computationally predicted evidence (provided with a confidence score to assess the reliability) from PubMed literature and VIRmiRNA,^52^ VmiReg,^53^ HumanViCe,^54^ RepTar,^55^ and Zikv-CDB^56^ databases.

### Metabolite-protein interaction resources

Metabolites are small molecules produced as intermediate or end products of microbial and host metabolic reactions, influencing immune homeostasis, host energy production, and signal transduction. Specific metabolites (such as bile acids, short-chain fatty acids, and tryptophan metabolites) are likely to play a key role in IBD pathogenesis.^57^^,^^58^ However, these have yet to be well defined. Metabolite-protein interaction resources integrating metabolomics and proteomics data can deepen our understanding of these interactions. Compared to the PPIs, the investigation of metabolite-protein interaction is more complicated and limited. The diversity of metabolites and the extremely short interaction duration, coupled with metabolomics technology limitations, pose challenges.^59^ MetalinksDB^60^ (https://metalinks.omnipathdb.org/) is an open-source database of intercellular metabolite-protein interactions, integrating various databases, such as STITCH,^61^ HMDB,^62^ and Recon3D,^63^ greatly extending coverage compared to previous resources. In addition, it provides biological annotations about pathways, diseases, and tissues that can be analyzed in the context. MiMeDB^64^ (https://mimedb.org/) is a multi-omics microbiome resource, linking microbes, microbial genomes, metabolites, and human exposome. Combining metabolite-protein interactions from MetalinksDB and microbial metabolomics data from MiMeDB investigates in detail the molecular mechanisms of microbial metabolites on host proteins. The enriched annotations help to understand these interactions within different biological contexts and diseases.

One specific type of metabolite-protein interaction that could yield fruitful insights in IBD is microbial metabolite interactions with G protein-coupled receptors (GPCRs). Microbial metabolites are known to target various GPCRs, highly expressed in the GI tract, affecting epithelial and immune cell populations.^65^ Dysregulated GPCR signaling by microbial metabolites contributes to IBD pathology, including increased intestinal permeability, immune activation, and chronic inflammation.^66^ High-throughput GPCR screens, like PRESTO-Salsa, evaluated numerous (>300) GPCRs *in vitro**^65^* and screened 1,041 metabolites to create an atlas of microbiota metabolome-human GPCR interactions. These data are publicly available (http://palmlab.shinyapps.io/presto-salsa/) and can inform connections between gut microbiota-derived metabolites, GPCRs, and downstream host signaling.

### Tools to explore host-microbe interactions

**Figure 2:**
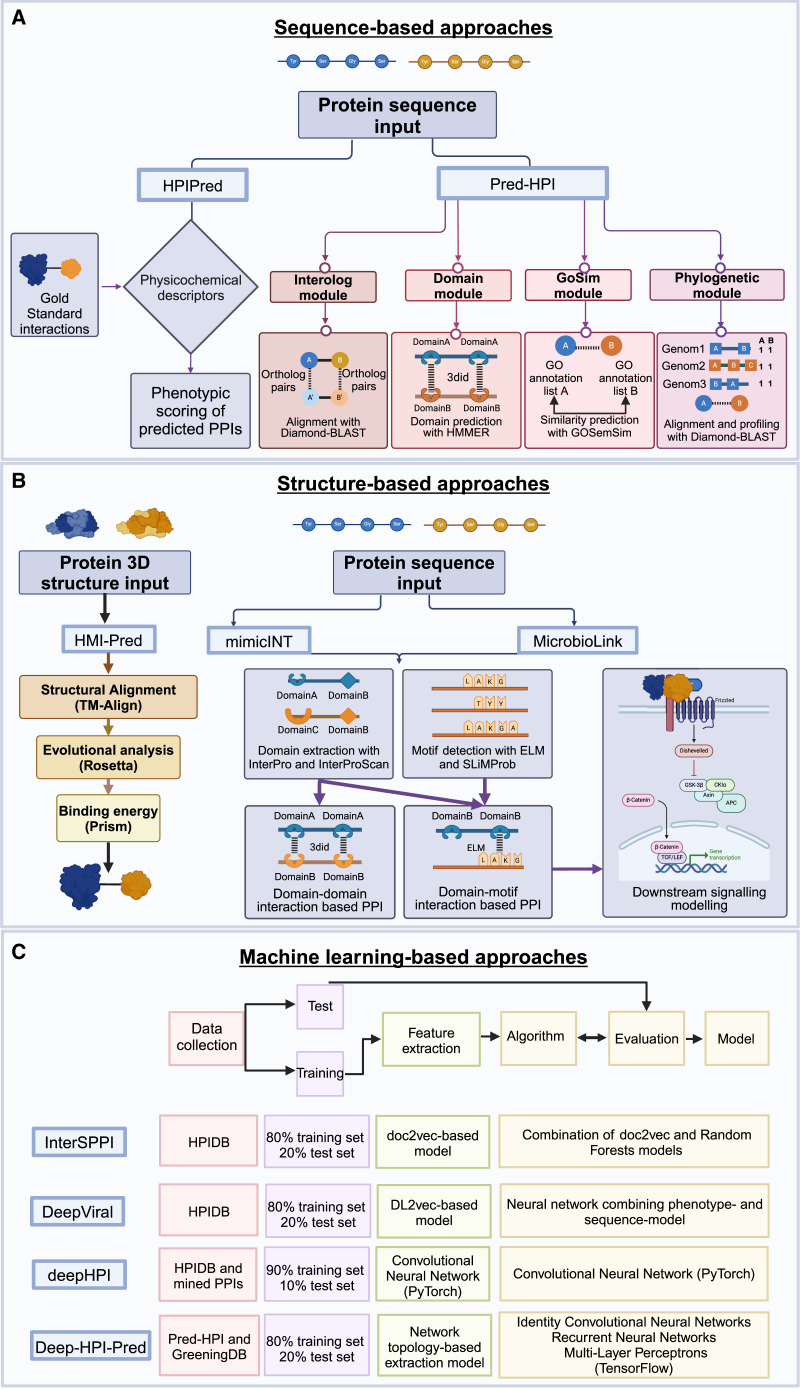
Comparison of *in silico* protein-protein interaction prediction tool detailing (A–C) (A) Sequence-based, (B) structure-based, and (C) machine-learning-based approaches. The figure was created by BioRender.

Various computational tools have been developed to explore host-microbe interactions, sub-classified into three categories according to the prediction strategies: sequence-based, structural-based, and machine-learning-based methods (Figure 2; Table 2). Each category employs different approaches to integrate omics data, facilitating our understanding of the gut microbiome’s impact on the host. Here, we highlight recent tools that employ these methods to predict the HMIs.

**Table 2:** Protein-protein interaction prediction tools

	*Availability*	*Function*	*Input*	*Output*
**Sequence-based approach**				

*HPIPred*	R package	Host-pathogen PPI prediction integrating phenotypic data	Proteome ID, descriptors, false discovery rate, model agreement (%)	Predicted interactome ranked by biological relevance
*Pred-HPI*	Web server	Predicting host-pathogen PPIs using four modules (interolog, domain-based, Gene Ontology similarity, phylogenetic profiling)	Protein sequences (FASTA)	Predicted interactome, visualized network

**Structure-based approach**				

*mimicINT*	Python and R packages	Inferring microbe-human PPIs using host-like elements and interaction templates from experimentally identified interaction templates	Protein sequences (FASTA)	Predicted interactome, functional enrichment analysis
*HMI-PRED 2.0*	Web server	Predicting host-microbe PPIs by interface mimicry	Protein structure (mmCIF/PDB) or PDB ID, host template (optional)	Predicted interactome, visualized network, enrichment analysis
*MicrobioLink*	*In silico* pipeline in Python	Integrating multi-omic data to discover bacterial impact on human proteins and downstream signaling	Proteins (UniProt/Gene names), differentially expressed genes (optional)	Predicted interactome, perturbed downstream signaling network, enrichment analysis

**Machine-learning-based approach**				

*InterSPPI and HVPPI*	Web server	Predicting human-bacteria/virus PPIs using sequence embedding	Protein sequences (FASTA)	Predicted interactome with probability score
*DeepViral*	*In silico* pipeline in Python	Predicting human-virus PPIs using deep learning and disease phenotypes	Protein sequences, feature embeddings	Predicted interactome, functional annotation
*deepHPI*	Web server	Predicting host-pathogen PPIs using convolutional neural networks	Protein sequences (FASTA), PPIs list (optional)	Predicted interactome with probability score, network visualization
*Deep-HPI-pred*	Web-based R/Shiny app	Predicting host-pathogen PPIs using network-driven feature learning method	Protein sequences (FASTA), training dataset (optional)	Predicted interactome, probability score, network visualization, enrichment analysis
*netMHC and panMHC*	Web server	(Pan-specific) binding of peptides to MHC class I and II molecules	Protein sequences (FASTA), peptide length	Detailed information on peptide binding to MHC molecules, such as residue position, binding affinity, binding core reliability, binding orientation, and classification as strong or weak binders, along with additional annotations and scoring metrics
*Graph-BERT model*	*In silico* pipeline in Python	Encoding PPI network graph with sequence-based features and learning the hidden representation of the feature vector for each node	Protein sequences (FASTA)	Predicted interactome

### Host-microbe PPIs

#### Sequence-based PPI prediction tools

Sequence-based methods rely on protein sequences to predict how microbial proteins may interact with host proteins by integrating metaproteomics and proteomics data. HPIPred^67^ encodes protein sequences based on amino acid features and integrates phenotypic data to highlight biologically significant findings. Users can select a UniProt Proteome ID or upload data, adjusting parameters for preferred false positive rates and physicochemical descriptors, such as hydrophobicity index, isoelectric point, or alpha-helix propensity. Pred-HPI^68^ integrates four widely used modules for predicting host-pathogen PPIs.

(1) Interolog module: interaction prediction based on the conservation of interactions across different species, leveraging known interactions.
(2) Domain-based module: identifying PPIs by analyzing the presence of specific protein domains known to mediate interactions, using domain-domain interaction information.
(3) Gene Ontology (GO) similarity module: GO annotations are used to infer PPIs assuming that proteins with similar functions or cellular localizations are likely to interact.
(4) Phylogenetic profiling module: comparing the evolutionary profiles of proteins, the module identifies co-evolving protein pairs across different species.

The input is host and/or pathogen protein FASTA sequences, and it returns an HPI prediction table with an interactive network figure.

Sequence-based PPI prediction tools offer powerful approaches, but they depend on the accuracy and completeness of available protein sequence data, which are limited particularly in less-studied microorganisms. The approach also misses the contextual factors influencing protein interactions, such as post-translational modifications, subcellular localization, and dynamic changes in the protein environment. Finally, structural protein conformations, which are crucial for understanding the interfaces necessary for interactions, are often overlooked by these algorithms.^69^

#### Structure-based PPI prediction tools

Structural feature-based methods identify potential interacting proteins using known structural features, such as domain-domain, domain-motif interactions, and molecular mimicry to identify potential interacting proteins. By integrating metaproteomics/metataxonomics and proteomics/transcriptomics data, these tools help in understanding the structural details of physical interactions between host and microbial proteins.

Tools like mimicINT^70^ and HMI-PRED^71^ predict interactions based on molecular mimicry. Microbial proteins manipulate host signaling to benefit themselves by imitating the structure of the host without the need for global structural homology or high sequence identity.^71^^,^^72^ mimicINT detects short linear motifs (SLiMs) and host-like globular domains in microbial protein sequences, using experimentally identified interaction templates (3did,^73^ ELM^74^). The domain identification is carried out by the InterProScan^75^ using the information from the InterPro database, and the host-like SLiMs detection is conducted by the SLiMProb tool^76^ using the ELM database. mimicINT also performs functional enrichment analysis to identify the host pathways potentially affected by microbial proteins. To reduce the false positives, mimicINT implemented a sub-workflow that uses Monte Carlo simulations to evaluate the chance of a given SLiM appearing by chance in query sequences. HMI-PRED 2.0 predicts PPIs by screening 3D structures of the microbial proteins with host interfaces in the template database through TM-Align^77^; evolutionarily conserved interaction hotspots are optionally filtered by Prism and Rosetta^78^^,^^79^ to map microbial proteins to the complementary proteins, with a false positive rate of 18%. HMI-PRED 2.0 not only derives microbial protein structures from RCSB PDB^80^ but also leverages AlphaFold2^81^ to predict the protein structures, enriching the number of potential interactions. Users can search the pre-computed interactions with enrichment analysis and visualization.

MicrobioLink^82^ (https://github.com/korcsmarosgroup/MicrobioLink2) is an integrated pipeline (developed by the authors of this review) that not only predicts HMIs using structural feature-based methods but also infers the downstream effects of the microbiome on the host, providing valuable insight into how microbial proteins in a certain context are influencing cellular signaling pathways of the host. MicrobioLink combines metaproteomics and proteomics/transcriptomics data and predicts the HMI using domain-domain or domain-motif interactions based on the gold standard information from the DOMINE^83^ and ELM^74^ databases. Network diffusion tools, such as CARNIVAL^84^ and TieDIE^85^ are used to infer the downstream signaling networks that connect the host proteins affected by the microbiome to the target genes of interest based on publicly available molecular interaction databases, such as OmniPath^86^ and CollecTRI.^87^ MicrobioLink provides (1) a multi-layered network, connecting microbial proteins directly to host proteins and indirectly to differentially expressed genes through PPIs and transcriptional regulatory interactions, and (2) also a functional enrichment plot to highlight the potentially perturbed signaling pathways.

**Figure 3:**
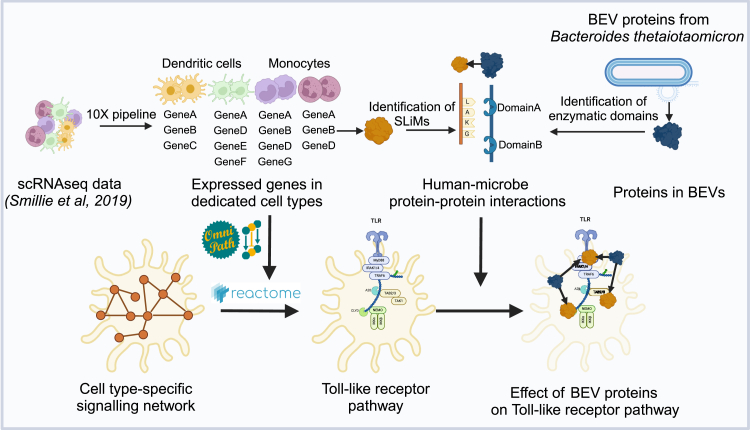
Workflow of MicrobioLink pipeline to predict the impact of bacteria on Toll-like receptor pathway in immune cells The figure was created by BioRender.

Demonstrating the pipeline’s effectiveness, we conducted a case study on bacterial extracellular vesicles (BEVs) from the commensal gut bacteria *Bacteroides thetaiotaomicron* (Bt)^88^ (Figure 3). In this study, we combined bacterial proteomics and single-cell RNA sequencing (scRNA-seq) from patients with IBD with the MicrobioLink pipeline to predict the effects of Bt BEVs on colonic immune cells, including dendritic cells, macrophages, and monocytes, focusing on the Toll-like receptor (TLR) pathway. Interestingly, we found that some proteins within BEVs target TLR pathway members in these immune cells differently depending on whether the individual is healthy or has UC. This suggests that BEVs might play a role in UC by targeting TLR pathways in a condition- and cell type-specific manner. Experimental validation confirmed that TLR4 and its adapter protein, TIRAP, are potential targets for BEV proteins. This study illustrates how combining multi-omics data can reveal complex host-microbe relationships with cell type-specific resolution and highlight the heterogeneity and importance of disease status for contextualizing these interactions.^88^

Structural PPI prediction methods rely on the availability and accuracy of known structural features, such as 3D structure or domain-domain and domain-motif interactions, which may not be fully characterized for all proteins. Similarly to the sequence-based approaches, these methods also miss context-specific interactions influenced by environmental conditions and localization. Accurate modeling of molecular mimicry is challenging, as slight structural alteration can lead to false predictions. Therefore, these methods should be complemented with other approaches and/or experimental validation to give a comprehensive understanding of host-microbe interactions.

#### Machine-learning-based PPI methods

Machine-learning (ML)-based methods handle high-dimensional, large-scale datasets to predict HMIs, integrating diverse omics data, including genomics, transcriptomics, proteomics, and meta-omics to build complex models.

InterSPPI^89^ is a random forest-based predictor of host-bacteria PPIs, using three conventional sequence-based encoding schemes and two host network property-related encoding schemes (NetTP and NetSS) to improve prediction accuracy. InterSPPI is developed based on the human-*Yersinia pestis* PPIs from HPIDB^32^ and PATRIC^90^ databases, but its performance has been evaluated and demonstrates strong robustness and generalizability. The input protein sequences must have at least 35 standard amino acids. The web server calculates the prediction scores of all possible sequence pair combinations, and it provides three optional specificity thresholds (0.95, 0.97, and 0.99). HVPPI^91^ predicts human-virus PPIs, developed by the same research group. Human-virus PPI data from HPIDB and SwissProt database were used to construct positive and negative data samples.

DeepViral^92^ is the first tool utilizing deep learning to combine phenotypes, functions, and taxonomies with protein sequences to predict human-virus interactions, leading to an improvement compared with approaches that focus only on protein sequences.

deepHPI^93^ is another deep-learning-based web server for predicting HPIs from protein sequence data. It provides four model types: plant-pathogen, human-bacteria, human-virus, and animal-pathogen, offering downloadable graphical visualizations, annotation information about protein node degree, GO terms, and links to public resources (UniProt, AmiGO, and NCBI). Compared to HVPPI (limited to three sequences per query), deepHPI can process 1,000 sequences in a single run. DeepHPI collected protein interaction data from HPIDB and extracted literature-validated PPIs and negative interaction data from the Negatome and Russell^94^ datasets, achieving higher accuracy of interaction prediction compared to HVPPI with an overall 95.73% to predict human-bacteria PPIs and 99.29% to predict human-virus HPIs.

Deep-HPI-pred^95^ uses a network-driven feature learning method to predict host-pathogen PPIs. Similar to deepHPI, it visualizes the results and provides GO analysis. It is the first application that offers users the autonomy to manually or automatically upload their training data. In addition, Deep-HPI-pred overcomes the limitations of previous approaches that did not fully utilize available structural and functional information.

The binding affinity of peptides/antigens to antibodies is crucial for understanding host-pathogen PPIs, particularly in the context of IBD, where these interactions influence the immune system’s ability to recognize and respond to microbial antigens contributing to disease pathogenesis. The affinity of this binding is vital for the stability and efficacy of the immune response and plays a role in maintaining gut homeostasis or contributing to inflammation.^96^ netMHC^97^ utilizes neural networks to predict peptide-binding affinities to major histocompatibility complex (MHC) class I molecules, facilitating the identification of microbial peptides that could provoke an immune response in patients with IBD. In addition, panMHC^98^ extends predictions to both MHC class I and II molecules, identifying peptides that can bind across various MHC alleles, which is particularly useful in understanding the diverse immune responses observed in IBD.

Large language models (LLMs) are also emerging as promising tools in the field. LLMs can process vast amounts of protein sequence data and predict interactions with high accuracy, potentially overcoming some limitations of traditional ML approaches. By leveraging the contextual understanding and pattern recognition strengths of LLMs, researchers can gain deeper insights into the complex web of protein interactions within and between species. For example, the Graph-BERT model^99^ predicts PPIs by classifying nodes in a graph as either interacting (positive label) or non-interacting (negative label). It employs the SeqVec language model to generate embeddings from protein sequences, addressing limitations of previous methods by overcoming issues like suspended animation and over-smoothing in graph neural networks. This approach significantly improves PPI prediction accuracy, leveraging advanced deep learning techniques for more accurate and robust results.

ML-based methods offer significant advantages in handling large-scale datasets and can reveal patterns among a high number of features that traditional methods may miss. However, they also face limitations: the primary challenge is the absence of experimentally verified negative training datasets,^93^^,^^100^ which are crucial for distinguishing non-interacting protein pairs from positive interactions. While databases like Negatome^101^ provide intra-species non-interacting protein pairs, there are few resources for inter-species PPI prediction, complicating the creation of comprehensive training sets. Moreover, ML models can be overfitted, particularly when trained on limited datasets, potentially leading to less generalizable predictions. While ML approaches can integrate diverse types of omics data, they often require extensive computational resources and expertise to develop and interpret the models.

### Host-microbe metabolite-protein interactions

Microbial metabolite-host protein interactions are an additional paradigm for modeling interspecies interactions. However, very few tools are currently available for modeling metabolite-protein interactions even on general level including host, microbe, and dietary-derived small molecules. One such tool is Causal Oriented Search of Multi-Omics Space (COSMOS),^102^ which identifies causal paths between metabolites, metabolic enzymes, and host transcription factors using at least two omics modalities from metabolomics, phosphoproteomics, and transcriptomics data. COSMOS leverages a meta prior knowledge network from the STITCH, OmniPath, and Recon3D databases as well as using a footprint-based approach, to infer interactions between proteins and transcription factors (kinase to kinase, TF to kinase, TF to metabolic enzymes, etc.), metabolites and enzymes (reactants to metabolic enzymes and metabolic enzymes to products), and metabolites and proteins (allosteric regulations). The recently published MRCLinkdb^103^ is a comprehensive resource and tool developed for analyzing metabolite-related ligand-receptor (ML-R) interactions in intercellular communication. It documents over 790 human and 670 mouse ML-R interactions and offers an interface for analyzing ML-R interactions using scRNA-seq data. Despite some limitations, such as not accounting for environmental factors, MRCLinkdb remains a valuable tool for exploring the role of metabolites in cellular communication.^103^

While these tools infer metabolite-protein interaction without considering the source (host, microbe, or diet), utilizing MiMeDB allows the selection of microbial metabolites. In combination with COSMOS or MRCLinkdb, these interactions can be interrogated to study gut microbial metabolite-host protein interactions.

### Omics integration within microbial or host compartments

While the resources and tools discussed so far relate to HMIs (i.e., vertical connections between the microbial and host compartments), resources and tools for horizontal connections (i.e., within each compartment) can enable the integration of additional omics layers, which can provide further insights into host-microbial relationships (Figure 1).

One principal example, within the microbial compartment, is the integration of metagenomics and metabolomics modeling. The Constraint-Based Reconstruction and Analysis (COBRA)^104^ method provides a systems biology framework for studying and simulating the metabolic networks of organisms at a genome-scale level. Using AGORA2 (assembly of gut organisms through reconstruction and analysis version 2), which contains the genome-scale metabolic reconstructions of 7,302 human gut microorganisms, COBRA generates metabolic network models from metagenomic data in which the nodes represent metabolites and edges represent biochemical reactions. Using flux balance analysis, the flow of metabolites through the network under different conditions can also be predicted. Thus, this methodology would enable investigators to predict *in silico* the metabolic output of a given metagenome, which can then be subsequently utilized to model gut metabolite-host signaling interactions. Alternatively, the developers of AGORA2 recently demonstrated that such *in silico* modeling in combination with *in vivo* microbiome-metabolome association studies can help to identify likely causal microbiome-metabolite association.^105^ These prioritized metabolites can then be utilized for further downstream modeling in the host.

On the human host side, linking patient genotype with host signaling is another area of interest, especially in the context of IBD where multiple SNPs are thought to perturb gene expression and impact host PPIs. More than 300 IBD-associated SNPs from multiple genome-wide association studies (GWASs) have been cataloged in the EBI GWAS Catalog.^106^ Additionally, resources such as the UK Biobank,^107^ the UK IBD BioResource,^108^ and BioMe^109^ contain genotype data from 1000s of patients with IBD. Several methods have recently been generated to try to harness knowledge of patient genotype to predict their impact on PPI networks and host signaling pathways such as iSNP^110^ and SNPsea.^111^ By harnessing these tools and databases, genetic variants that influence host-microbe interactions could be revealed, which is a major missing gap in our current understanding of IBD pathogenesis.^112^

Another resource that facilitates omics integration in either the host or microbial compartment in IBD, specifically of a single modality from multiple studies, is the Inflammatory Bowel Disease Transcriptome and Metatranscriptome Meta-Analysis (IBD-TaMMA) platform.^113^ This platform applies batch correction and a standardized analysis pipeline to synthesize data from multiple transcriptomics and metatranscriptomics studies in IBD, thereby addressing the limitations of weak statistical power and experimental variability of individual studies. In doing so, it provides a comprehensive overview of publicly available datasets from patients with IBD and controls, yielding more robust insights into the changes in host gene expression from either blood or intestinal tissues and gut microbial composition in patients with IBD compared to controls.^114^

### Challenges and future directions for host-microbiome research in IBD

Integrating high-throughput data from various biological levels could provide a more comprehensive picture of the complex interplay between the host and gut microbiota in IBD. However, combining multi-omics data presents significant challenges.^115^

In terms of the microbiome compartment, a precise definition of the “IBD-associated microbiome” remains elusive due to significant inter-personal variations and lack of specific microbial signatures associated with IBD subtypes.^116^ Additionally, spatial heterogeneity presents challenges in understanding the microbial composition and function along the GI tract. Current multi-omics approaches often lack the resolution to capture these spatial variations, potentially overlooking important microbial niches relevant to IBD pathogenesis. Recently developed methods for mucosal metatranscriptomics provide insights into these niches,^117^ but temporal heterogeneity and diet are also important factors that influence the detected microbiome composition.^115^^,^^118^ Future studies should carefully consider sample timing, patient diet, and other potential covariates to ensure data accuracy and generalizability. The impact of altered metabolite and microbe composition on the microbial community itself remains uncharacterized. The lack of knowledge on microbe-microbe and microbe-metabolite interactions challenges understanding the connection between dysbiosis and gut inflammation.^116^

In the host compartment, acquiring gut tissue specimens is logistically challenging and requires coordination between clinicians and researchers. Most multi-omics studies in IBD lack single-cell resolution, which is required for a more granular understanding of HMIs,^115^ while single-cell transcriptomics could reveal heterogeneity in cell populations within the gut and their interactions with the microbiota, providing deeper insights into IBD pathogenesis. Furthermore, clinical metadata, such as age, sex, disease severity, and medication history, must be meticulously recorded for each patient.

From a technology point of view, there are relatively few metaproteomics studies^119^^,^^120^^,^^121^ in IBD compared to other omics techniques, due to technical complexity and novelty of the field compared to genomics or transcriptomics.^121^ In contrast, although metabolomics studies are more prevalent in IBD research, very few tools and resources are currently available to accurately model gut metabolite-host protein interactions. Therefore, further alignment between specific omics modalities and computational analysis tools is required. Multi-omics data are usually generated through diverse platforms and technologies resulting in heterogeneity between studies leading to a bias in data interpretation.^115^ Standardized protocols for data collection, processing, and storage are crucial for robust multi-omics analyses. Advanced computational tools and statistical methods are required to handle the high dimensionality and complex relationships within multi-omic data, moving beyond simple correlation methods. Despite these challenges, the field is evolving with novel strategies, such as ML algorithms, which can handle data heterogeneity and high dimensionality leading to a more comprehensive understanding of HMIs in IBD.^115^ Initiatives and open-access platforms are increasing for sharing multi-omics datasets and promoting data standardization.^122^ Furthermore, integrating single-cell and spatial multi-omics data can provide a more detailed understanding of cellular heterogeneity and microbiota interactions, leading to more targeted personalized treatment approaches.^123^

An exciting future direction in IBD HMI research is the identification of therapeutic targets within the GPCR family, crucial for mediating responses to microbial signals in the gut, influencing inflammation and immune responses, and are also one of the most common targets of all Food and Drug Administration-approved drugs.^66^ Combining multi-omics data with network biology approaches could unveil novel therapeutic targets in IBD.^66^

Building on the identification of therapeutic targets, future focus should include drug-microbiome-host and diet-microbiome-host modeling to optimize treatment strategies. Drug-microbiome-host modeling is critical to understand how treatments, like 5-aminosalicylate agents, are metabolized by the microbiome before activation,^123^ facilitating the prediction and optimization of therapeutic outcomes. Integrating drug response data with multi-omics datasets enables precise predictions of therapeutic responses, improving treatment strategies.^124^ Similarly, diet-microbiome-host modeling tailors dietary interventions based on personalized microbiome profiles, enhancing gut health and suppressing inflammation, offering a more natural approach for IBD management.^125^^,^^126^

Finally, multi-omics studies need to reveal disease mechanisms, validated through *in vitro* and/or *in vivo* experiments, such as patient-derived organoids and co-culture systems.^127^^,^^128^^,^^129^ Additionally, the unique capabilities of *in vivo* murine models of colitis should not be disregarded in the current era of omics, big data, and precision medicine.

Acknowledging and addressing these challenges can unlock the full potential of multi-omic data in IBD research. Upcoming multi-omics studies will generate vast data, standardized protocols, in-depth capturing of patient clinical metadata, and advanced computational tools and statistical analysis to unravel host-microbial relationships underpinning IBD pathogenesis. These efforts are urgently needed to overcome the therapeutic ceiling in IBD and improve patient outcomes.

### Conclusion

While the gut microbiome remains a central facet of IBD pathogenesis, the precise HMIs contributing to IBD pathogenesis remain largely unknown. Multi-omics profiling of both the microbiome and host compartments in patients with IBD holds great promise for gaining a holistic understanding of the HMIs contributing to IBD pathogenesis, which may not be captured through single-omics analysis alone. However, until now, limited mechanistic insights have been yielded due to the inherent complexities of multi-omics data integration. In this review, we have summarized state-of-the-art systems biology tools and resources that have yet to penetrate the field of IBD but could be harnessed to identify more granular host-microbial interconnections from high-dimensional multi-omics datasets by modeling protein-protein, RNA-RNA, and metabolite-protein interactions. As the field continues to evolve, ongoing efforts to standardize data collection and analysis protocols, along with the establishment of open-access platforms for data sharing, will be crucial to ensure the reliability and reproducibility of multi-omics data interpretation. These exploratory analyses should be followed by *in vitro* and *in vivo* experimental approaches to validate and further characterize biological signals captured through systems biology analysis of multi-omics datasets. By addressing current limitations, the multi-omics era promises to reveal new perspectives in IBD and other microbiome-associated complex diseases and has exciting potential to inform the development of much needed precision medicine strategies.

## Acknowledgments

J.P.T. is supported by the Chain-Florey Clinical PhD Fellowship jointly funded by the 10.13039/501100000272National Institute for Health Research (NIHR) 10.13039/501100013342Imperial Biomedical Research Centre (BRC) and the 10.13039/100014013UKRI Medical Research Council (MRC) Laboratory of Medical Sciences (10.13039/501100000608LMS). The work of T.K. and L.G. was supported by a 10.13039/100014013UKRI BBSRC Institute Strategic Program Food Microbiome and Health BB/X011054/1 and its constituent project BBS/E/F/000PR13631. T.K. was supported by the 10.13039/501100013342NIHR Imperial Biomedical Research Centre Organoid Facility. The views expressed are those of the authors and not necessarily those of the NIHR or the UK Department of Health and Social Care.

## Author contributions

Y.Z. collected the data, drafted the manuscript, and prepared the figures and tables with the help of L.G. J.P.T. and L.G. drafted the manuscript with input from T.K. T.K. and L.G. supervised the work and developed the final manuscript.

## Declaration of interests

The authors declare no competing interests.
